# Beyond Chemical Triggers: Evidence for Sound-Evoked Physiological Reactions in Plants

**DOI:** 10.3389/fpls.2018.00025

**Published:** 2018-01-30

**Authors:** Jihye Jung, Seon-Kyu Kim, Joo Y. Kim, Mi-Jeong Jeong, Choong-Min Ryu

**Affiliations:** ^1^Molecular Phytobacteriology Laboratory, Infectious Disease Research Center, Korea Research Institute of Bioscience and Biotechnology, Daejeon, South Korea; ^2^Department of Biological Sciences, Korea Advanced Institute of Science and Technology, Daejeon, South Korea; ^3^Personalized Genomic Medicine Research Center, Korea Research Institute of Bioscience and Biotechnology, Daejeon, South Korea; ^4^National Institute of Agricultural Science, Rural Development Administration, Wanju, South Korea

**Keywords:** plant protectant, plant stimulant, sound vibration, transcriptome, physical trigger, ripening delaying

## Abstract

Sound is ubiquitous in nature. Recent evidence supports the notion that naturally occurring and artificially generated sound waves contribute to plant robustness. New information is emerging about the responses of plants to sound and the associated downstream signaling pathways. Here, beyond chemical triggers which can improve plant health by enhancing plant growth and resistance, we provide an overview of the latest findings, limitations, and potential applications of sound wave treatment as a physical trigger to modulate physiological traits and to confer an adaptive advantage in plants. We believe that sound wave treatment is a new trigger to help protect plants against unfavorable conditions and to maintain plant fitness.

## Introduction

Sound is an omnipresent feature throughout the world (Theunissen and Elie, 2014). The definition of sound is “a vibration that typically propagates as an audible wave of pressure, through a transmission medium such as a gas, liquid or solid,” and each sound is characterized by its wavelength hertz (Hz), intensity (decibel), speed, and direction (Shipman et al., 2012). The audible sound that is perceptible by humans has frequencies from about 20 to 20,000 Hz, and above it is ultrasonic. In air at standard temperature and pressure, the corresponding wavelengths of sound waves range from 17 m to 17 mm. The speed of sound depends on the medium the waves pass through, and is a fundamental property of the material (McCall, 2010). Living organisms produce and perceive sound to help understand the environment around them (Morales et al., 2010; Aggio et al., 2012). Sound-based communication through the eardrum or specialized mechanosensory systems are commonly found in humans and certain terrestrial mammals (Grothe et al., 2010). Even insects emit species-specific sounds to help them escape unfavorable conditions or to attract mate (Djemai et al., 2001). Moreover, fruit flies, snakes, frogs, and birds can perceive sound vibrations without an eardrum (Gagliano et al., 2012). Fruit flies detect vibrations via their antennae, whereas snakes use their jawbones (Gagliano et al., 2012). Plants perceive sound using an unidentified organ. Unlike wind, sound also has a frequency. This plays a critical role in the impact of sound on living organisms. Although the role of sound in the animal kingdom has been studied, how plants (as sessile organisms) respond to sound has not been extensively elucidated due to the lack of an organ in plants designed to recognize air vibrations, like eardrums in humans. However, a growing body of evidence emerging from biological studies on the response of plants to sound waves indicates that plants are highly sensitive organisms that generate and react to sound signals from their environment (Mishra et al., 2016). Previously, farmers and several scientists in China and South Korea applied music referred to as “Green Music” to plants in order to improve plant health and yield (Qin et al., 2003). In these trial experiments, the results were sometimes inconsistent and variable in different locations. In addition, the sound used in these experiments was not standardized, and was not performed on the uniform and consistent hertz (vibration) and decibel (strength) levels of the signals, and these studies utilized different styles of music for sound treatment (Qin et al., 2003). Therefore, studies involving the use of sound as a trigger have been recognized as fringe science. However, recent findings using cutting-edge technology, quality control for hertz and decibel levels, and the integration of big data have helped change the viewpoint about this field as it has entered the realm of generally accepted science (Gagliano et al., 2012; Chowdhury et al., 2014; Mishra et al., 2016). We now believe that plants can indeed benefit from sound through their mechanosensory machinery. Many studies have already demonstrated sound-induced phenotypic changes and possible sound signaling pathways in model and crop plants. In this review, we discuss how plants generate and respond to sound and how sound can be used to improve plant growth and plant resistance against biotic and abiotic stresses. Here, we propose that sound is an emerging physical trigger in plants beyond chemical triggers, such as plant hormones and other immune activators which have been used to improve plant health.

## Production (Speaking) and Perception (Listening) of Sound in Plants

To understand how plants respond to sound, we need a new framework beyond chemical compound-based signal initiation and responses in plants. We therefore classified the steps involved in this multi-layered process from the emission of sound by plants to the altered phenotypes observed after the plant has recognized the sound information. This basic knowledge helps us elucidate how sound signals trigger changes in plants in nature.

### Can Plants Make Sound Vibration?

**FIGURE 1:**
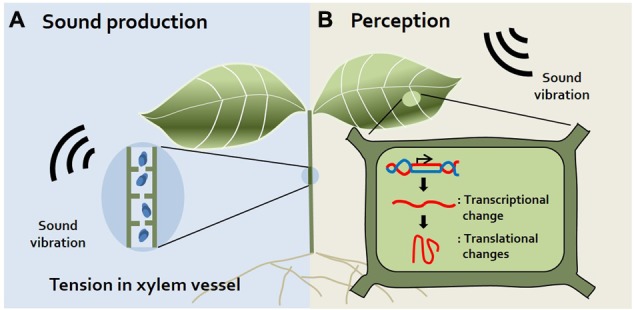
Sound production and perception in plants. **(A)** Sound production. Plants produce sound vibrations in their xylem via the generation of tension in the xylem vessel when its diameter decreases (Hölttä et al., 2005). Additionally, gas bubbles produced in xylem vessels during transpiration may produce sound (Laschimke et al., 2006). **(B)** Sound Perception. Although there are no visible alterations, transcriptional and translational changes occur in plants exposed to sound vibrations. Levels of mechano-stimulus responsive, signaling-related, redox homeostasis, and defense-related transcripts are changed in sound-exposed plants (Ghosh et al., 2016). However, the specific organs or proteins used for sound perception have not yet been identified.

It was long thought that plants do not make sounds. Although humans cannot perceive sound from plants, recent studies using small, highly sensitive sound receivers have surprisingly demonstrated that plants indeed make spontaneous sounds and even release sound emissions from their xylem (Borghetti et al., 1989; Ritman and Milburn, 1990; Laschimke et al., 2006) (**Figure 1A**). Since the xylem is a water-transporting system in plants, transpiration, and re-hydration occur in xylem vessels. Transpiration produces tension in xylem vessels, and simultaneously, gas bubbles (cavitation) are produced in xylem vessels during transpiration. Indeed, gas bubbles adhering to vessels may produce sound in plants (Laschimke et al., 2006). It is reported that when transpiration decreases, audible sound is released and transpiration increases, ultrasonic emission is released (Ritman and Milburn, 1990). Also, the fact that plants emit ultrasonic vibration has been disputed, but it has recently been confirmed that ultrasonic vibration of 20–105 kHz is emitted by connecting a sensor directly to the plant stem that has been barked (Laschimke et al., 2006). Moreover, sound vibrations are generated when the diameter of the xylem vessel decreases (Hölttä et al., 2005). Increasing studies also suggest that tension in the xylem is the cause of this sound in plants. However, whether plants use this ultrasonic sound for their communication remains to be elucidated. In addition to sound produced by plants, the idea that insects also produce sounds is widely accepted because we often hear sounds such as bees buzzing, insects chewing, and flies buzzing. How do the sounds of insects affect plants? Specific frequencies of bee buzzing facilitate the pollination of flowers, since these sounds induce the release of pollen from plant anthers (De Luca and Vallejo-Marin, 2013). In addition, insect chewing serves as an alarm signal to plants. Recorded insect chewing sounds induce the production of chemicals related to plant defense in *Arabidopsis*, such as glucosinolate and anthocyanin (Appel and Cocroft, 2014). Collectively, these findings suggest that plants respond to insects through sound, sometimes serving as warning signals or beneficial signals to the plant.

### Can Plants Respond to Sound?

How can plants perceive sound and thereby respond to specific stress stimuli without a hearing organ? The roots of *Zea mays* were reported to bend toward sound with a frequency of 100–300 Hz among the tested frequencies of 0–900 Hz in the hydroponic system (Gagliano et al., 2012), indicating that sound induces structural responses in plants. Even small environmental stimuli such as touch or wind alter the transcriptional levels of plants. A recent study described commonalities and differences between responses to sound and mechanical vibrations at the gene expression level. Expression of some genes (e.g., *MSL* and *MCA*) encoding mechanosensitive ion channels, which may recognize mechanical signals, was reported to differ between sound-exposed and touch-treated *Arabidopsis* plants (Ghosh et al., 2017). This supports the notion that sound vibrations provide a special stimulus to plants, unlike mechanical vibrations. In addition, sound vibration increased the rate of growth by changing the cell metabolism of yeast, but reduced biomass production. Theses result imply that sound affects the cell level rather than the specific structure of the organism (Aggio et al., 2012). Here, we focus on recent findings about plant responses to sound treatment based on transcriptome and proteomics technology (**Figure 1B**).

Although sound is not a visible or chemical stimulus, plants exposed to sound (a physical force) produce increasing amounts of mRNA (Ghosh et al., 2016), suggesting that sound induces changes in plants at the transcriptional level. Indeed, two genes, the fructose 1,6-bisphosphate aldolase (*ald*) and Rubisco small sub-unit (*rbcS*) genes, which play critical roles in photosynthesis, were specifically induced in rice following 125 and 250 Hz sound treatment (Jeong et al., 2008) (**Table 1**). Continuous exposure to sound is thought to enhance plant growth by promoting CO_2_ fixation (Uematsu et al., 2012). These findings can be attributed to sound-mediated photosynthesis-related gene expression and increased CO_2_ fixation. A similar study showed that the expression of genes in the Gene Ontology categories mechano-stimulus responsive, signaling related, redox homeostasis, biosynthesis, and defense increased in response to exposure to 500 Hz sound waves in *Arabidopsis* (Ghosh et al., 2016) (**Table 1**). These results imply that sound vibrations provide a stimulus to plants. More extensive research is needed on the function of the identified genes and the signaling network. Many questions still remain to be answered, such as “which part of the response is specific to sound (e.g., how does the response differ according to the sound)?” and “Can plant recognize the sound and other mechanical signals differently?”

**Table 1:** Responses of plants to sounds of different frequencies and magnitudes.

Plant species	Plant responses	Sound-exposed tissues	Frequency (Hz)	Magnitude (dB)	Duration	Reference
*Arabidopsis*	Increased expression of defense-related genes	Shoot	500	80	1 h	Ghosh et al., 2016
	Increased expression of mechano-stimulus responsive genes	Shoot	500	80	1 h	Ghosh et al., 2016
	Increased expression of photosynthesis-related proteins and genes	Shoot	250 and 500	80	1 h	Kwon et al., 2012
	Increased expression of redox homeostasis genes	Shoot	500	80	1 h	Ghosh et al., 2016
Cotton	Increased yield	Shoot	100–1000	70	3 h (every other day)	Hassanien et al., 2014
Cucumber	Increased yield	Shoot	100–1000	70	3 h (every other day)	Hassanien et al., 2014
Chrysanthemum	Changes in hormone levels	Mature callus	1400	95	1 h	Bochu et al., 2004
	Increased levels of soluble proteins	Stem	1000	100	1 h for 6 and 9 days	Yi et al., 2003
Lettuce	Increased yield	Shoot	100–1000	70	3 h (every other day)	Hassanien et al., 2014
Maize	Root tip bending	Root	100, 200, and 300	Unknown	Unknown	Gagliano et al., 2012
Pea	Root growth toward flowing water	Root	Unknown	Unknown	Unknown	Gagliano et al., 2017
Rice	Increased expression of light responsive genes	Shoot	125 and 250	65–70	4 h	Jeong et al., 2008
	Increased yield	Shoot	100–1000	70	3 h (every other day)	Hassanien et al., 2014
	Enhanced tolerance to drought stress	Shoot	800–1000	100	1 h	Jeong et al., 2014
	Increased photosynthesis	Shoot	800–1000	100	1 h	Jeong et al., 2014
Spinach	Increased yield	Shoot	100–1000	70	3 h (every other day)	Hassanien et al., 2014
Strawberry	Increased photosynthesis	Shoot	Unknown	Unknown	3 h (every day)	Qi et al., 2009
Sweet pepper	Increased yield	Shoot	100–1000	70	3 h (every other day)	Hassanien et al., 2014
Tomato	Increased yield	Shoot	100–1000	70	3 h (every other day)	Hassanien et al., 2014
	Delayed ripening	Fruit	1000	100	6 h	Kim et al., 2015
Wheat	Increased yield	Shoot	100–1000	70	3 h (every other day)	Hassanien et al., 2014

In fact, plant hormone signaling networks are already beginning to be elucidated. Distinct and dynamic changes in plant hormones and the downstream signaling cascades are known to occur throughout a plant’s lifecycle. Plant hormones typically regulate plant cellular processes and orchestrate most aspects of plant physiology including plant growth, flowering, ripening, senescence, and defense responses (Hou et al., 2009; Qi et al., 2009; Hassanien et al., 2014; Kim et al., 2015). Recent studies showed that, in *Arabidopsis*, treatment with 500 Hz sound induces the production of the growth-related hormones indole-3-acetic acid (IAA) and gibberellin (GA) 3 and the defense-related hormones salicylic acid (SA) and jasmonic acid (JA) (Ghosh et al., 2016). Increased IAA levels and reduced abscisic acid (ABA) levels were also detected in *Chrysanthemum* exposed to a 1.4 kHz sound stimulus (Bochu et al., 2004) (**Table 1**). Although the optimal sound treatment differs depending on the plant species, such sound-induced hormonal changes might increase plant growth and provide strong resistance against biotic or abiotic stress. A recent study reported that plant roots can respond to environmental sound (Gagliano et al., 2017) (**Table 1**). Specifically, *Pisum sativum* roots locate water by actively growing toward flowing water belowground (Gagliano et al., 2017). This implies that plants also respond to natural sound in the environment.

## Application of Sound Waves to Improve Plant Health

As mentioned above, plants appear to perceive sound, as they exhibit transcriptional and hormonal changes in response to sound wave treatment. Next, we provide an overview of the implications of sound wave treatment in the field or growth room. Recent studies using ‘omics’ technologies, such as transcriptome and proteomic analyses, showed that proper sound treatment has a positive effect on plant growth. Based on this information, we discuss the expansion of the use of sound in modern agriculture and plant biology.

### Plant Protectants

**FIGURE 2:**
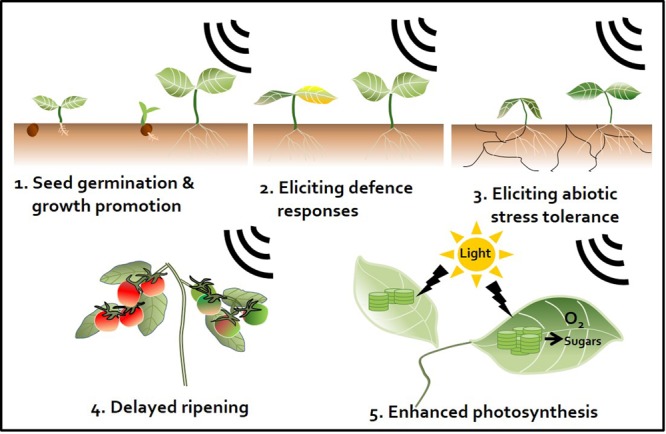
Sound waves as a plant stimulant and protectant. Artificial sound treatment can elicit various effects in plants. First, enhancement of seed germination and plant growth. Sound promotes plant growth by regulating the plant growth hormones indole-3-acetic acid (IAA) and gibberellin (Bochu et al., 2004; Ghosh et al., 2016). Second, induction of plant defense responses against pathogens. Sound pretreatment enhances plant immunity against subsequent pathogen attacks by activating the plant defense hormones salicylic acid (SA) and jasmonic acid (JA) (Hassanien et al., 2014; Ghosh et al., 2016). Third, induction of abiotic stress tolerance. For instance, sound treatment triggers drought tolerance by changing the elasticity and flexibility of the cell wall, which affects the ability of plants to absorb water (Jeong et al., 2014). Fourth, perturbation of ripening. Sound treatment disrupts the ripening of tomato fruit. Ethylene production is delayed by down-regulation of ethylene biosynthesis and expression of signaling-related genes (Kim et al., 2015). Fifth, enhancement of the photosynthetic capacity. Sound treatment increases expression of photosynthesis-related genes, such as those encoding fructose 1,6-bisphosphate aldolase and the rubisco small sub-unit, and may induce CO_2_ fixation (Jeong et al., 2008; Uematsu et al., 2012).

Exposing plants to sound activates plant innate immunity and (more specifically) elicits representative SA and JA defense signaling pathways similar to those observed in response to different chemical triggers (Ghosh et al., 2016). Meta-analyses have demonstrated the occurrence of sound-mediated plant protection through the activation of the systemic immune response in crop plants such as pepper, cucumber, tomato, and strawberry (Hou et al., 2009; Chowdhury et al., 2014; Mishra et al., 2016; Choi et al., 2017) (**Figure 2**). The Ca^2+^ ions influx the cytosol from outside the plants membrane by 1000 Hz sound exposure. These ions might serve as secondary messengers upon exposure to environmental stress, thereby enhancing plant resistance against microbial pathogens. *Arabidopsis calmodulin-like 38* (*CML38)* gene, which encodes a calcium-binding messenger protein, is upregulated in response to sound treatment in *Arabidopsis* leaves (Ghosh et al., 2016). In addition, membrane architecture changes in response to sound treatment, which may facilitate the movement of signaling components related to defense responses (Mishra et al., 2016). In addition to biotic stress responses, sound treatment increases plant tolerance to abiotic stresses such as drought. For example, rice exposed to 0.8–1.5 kHz sound waves for 1 h showed increased tolerance to drought stress, with higher water contents and stomatal conductance than the control group (Jeong et al., 2014) (**Figure 2** and **Table 1**). Water deficiency is first detected in the plant root, and drought stress signaling is transmitted to the shoot through the xylem. Since membrane architecture changes in response to sound treatment, the plant is better able to absorb water in situations where water is lacking. From a hormonal perspective, crosstalk between ABA and JA regulates the response to drought (Riemann et al., 2015). Furthermore, among hormones, ABA is the most important regulator of the plant response to abiotic stress, especially osmotic stress (Kim et al., 2010). Consequentially, sound waves may be involved in both abiotic and biotic stress responses through the regulation of various plant hormones.

### Post-harvest Delaying Agent

Fruit ripening is associated with dramatic increases in ethylene production after harvest. Reducing ethylene production is an important way to delay fruit ripening. We previously showed that sound-treated tomato showed reduced ethylene production and delayed softening compared with the control (Kim et al., 2015) (**Figure 2**). The expression of ethylene biosynthesis genes *ACS2, ACS4, ACO1, E4*, and *E8* and ripening-related genes *RIN, TAGL1, HB-1, NOR*, and *CNR* was delayed in tomato treated with 1 kHz sound versus the control (Kim et al., 2015). The expression of genes encoding transcription factors RIN and HB-1, which control the expression of ethylene-related genes, was also affected in tomato treated with sound stimuli (Kim et al., 2016). Exposure to 1 kHz sound induces tomato fruit to remain firm for longer (Kim et al., 2015) (**Table 1**). Although the optimal sound conditions (frequency and decibels) must be determined depending on crop species, the use of sound wave treatment would be a convenient way to delay fruit ripening without the use of chemical preservatives or genetic modification. In addition to delaying fruit ripening, perhaps the quality and yields of post-harvest crops can be improved by sound treatment.

### Plant Growth Stimulants

Sound treatments have been broadly applied to alter plant growth. For example, sound-treated tomato showed 13.2% increased yields compared with the control (Hou et al., 2009). In contrast, high-frequency, high-decibel sound damages cells (Bochu et al., 1998). However, treatment with 5 kHz (92 dB) sound waves increased tiller growth and dry weight in wheat (Weinberger and Measures, 1979) (**Figure 2**). The result would be good to speculate not only on direct cellular mechanisms but also on indirect targets such as hormones and photosynthesis signaling while sound transduction pathway remains to be identified. Additionally, the improvement of plant growth by sound treatment has been studied in many crops such as chrysanthemum, sweet potato, cucumber, lettuce, spinach, cotton, rice, and wheat (Hassanien et al., 2014) (**Table 1**). However, the mechanism underlying how plant growth is improved by treatment with sound waves has not been intensively studied. A simple explanation for this effect is that this treatment alters the levels of plant growth regulatory hormones. As mentioned earlier, sound exposure alters endogenous hormone levels in plants. Increased IAA and decreased ABA levels in response to sound exposure may be the major factors underlying the effect of sound waves on promoting plant growth. Other studies have shown that the levels of soluble proteins and soluble sugars increase in response to sound treatment (Yi et al., 2003) (**Table 1**). Soluble sugars can also be a factor in promoting plant growth, as they can serve as an energy source. In addition, although the proper frequency of sound differs depending on plant species, a number of molecular studies support the notion that sound also induces plant growth promotion and seed germination. Of the possible mechanisms underlying the plant growth-promoting effects of sound treatment, the enhancement of photosynthesis represents a strong candidate for further characterization (**Figure 2**). Increased photosynthetic ability has been observed in strawberry and rice in response to sound treatment (Qi et al., 2009; Meng et al., 2012; Jeong et al., 2014) (**Table 1**). Proteomics analysis showed that photosynthesis-related proteins were highly expressed at 8 h after 250 or 500 Hz sound exposure in *Arabidopsis* (Kwon et al., 2012) (**Table 1**). Since sound energy induced secondary products can make chemical energy, sound treatment is thought to improve photosynthesis (Meng et al., 2012). These findings suggest that sound treatment can improve the quality of vegetable and fruit crops.

## Perspectives and Remaining Questions

Sound represents a potential new trigger for plant protection (Mishra et al., 2016). To date, the use of this new trigger has been introduced and validated in proof-of-concept studies for its potential applications to plant biology. However, there are limitations to this treatment that must be overcome, and unanswered questions remain to be explored. Here, we focused on sound waves as a stress reliever in plants. After summarizing previous findings, there are still some major concerns about the use of sound treatment in plant science. First, we still do not understand how the plant initially perceives sound, even though there is accumulating information about plant responses to different wavelengths of sound and the responses of different plant species. Without eardrums, how do plants physically recognize the strength and wavelengths of sound signals and integrate this information in plant cells? This issue is also critical from a practical viewpoint. The discovery of an organ or a specific protein in plants that recognizes sound waves would help us maximize the effectiveness of the use of sound treatment in field trials. Second, technology used to engineer sound quality, such as the fine turning, modification, and mixing of sounds, must also be improved to facilitate its use for sound-mediated stress relief and increased plant growth. Third, the analysis of plant biomarkers such as Pathogenesis-Related 1 protein (PR1) (for systemic acquired resistance) will help scientists optimize sounds to maximize sound-specific plant stress relief (van Loon, 1975). Fourth, we must be concerned about the side effects of sound waves as well. Humans can differentiate and recognize sounds ranging from 20 to 20,000 Hz. If the sound vibration used to treat plants causes damage to animals, humans, or microbes after long-term exposure, a detailed examination and evaluation of the effects of various exposure times and high-frequency (e.g., above 20,000 Hz) will be required. To minimize side effects from this treatment, different aspects of the responses of animals to the selected wavelength need to be assessed. In conclusion, the use of sound as a new plant trigger is in its infancy, but it has already shown great potential (Chowdhury et al., 2014). If the proper electric power supply, speakers, and associated sound-generating equipment are utilized, sound treatment can constitutively be applied for long periods of time without additional input. This unique setup, which has not been tested before, awaits your next experiment.

## Author Contributions

C-MR and JJ designed the review. JJ, S-KK, JK, M-JJ, and C-MR wrote the review.

## Conflict of Interest Statement

The authors declare that the research was conducted in the absence of any commercial or financial relationships that could be construed as a potential conflict of interest.

## References

[B1] Aggio R. B. M. Obolonkin V. Villas-Bôas S. G. (2012). Sonic vibration affects the metabolism of yeast cells growing in liquid culture: a metabolomic study. *Metabolomics* 8 670–678. 10.1007/s11306-011-0360-x

[B2] Appel H. M. Cocroft R. B. (2014). Plants respond to leaf vibrations caused by insect herbivore chewing. *Oecologia* 175 1257–1266. 10.1007/s00442-014-2995-6 24985883 PMC4102826

[B3] Bochu W. Jiping S. Biao L. Jie L. Chuanren D. (2004). Soundwave stimulation triggers the content change of the endogenous hormone of the *Chrysanthemum* mature callus. *Colloids Surf. B Biointerfaces* 37 107–112. 10.1016/j.colsurfb.2004.03.004 15342020

[B4] Bochu W. Yoshikoshi A. Sakanishi A. (1998). Carrot cell growth response in a stimulated ultrasonic environment. *Colloids Surf. B Biointerfaces* 12 89–95. 10.1016/S0927-7765(98)00061-7

[B5] Borghetti M. Raschi A. Grace J. (1989). Ultrasound emission after cycles of water stress in *Picea abies*. *Tree Physiol.* 5 229–237. 10.1093/treephys/5.2.229 14972990

[B6] Choi B. Ghosh R. Gururani M. A. Shanmugam G. Jeon J. Kim J. (2017). Positive regulatory role of sound vibration treatment in *Arabidopsis thaliana* against *Botrytis cinerea* infection. *Sci. Rep.* 7:2527. 10.1038/s41598-017-02556-9 28559545 PMC5449397

[B7] Chowdhury M. E. K. Lim H. Bae H. (2014). Update on the effects of sound wave on plants. *Res. Plant Dis.* 20 1–7. 10.5423/RPD.2014.20.1.001

[B8] De Luca P. A. Vallejo-Marin M. (2013). What’s the ‘buzz’ about? The ecology and evolutionary significance of buzz-pollination. *Curr. Opin. Plant Biol.* 16 429–435. 10.1016/j.pbi.2013.05.002 23751734

[B9] Djemai I. Casas J. Magal C. (2001). Matching host reactions to parasitoid wasp vibrations. *Proc. Biol. Sci.* 268 2403–2408. 10.1098/rsbp.2001.1811 11747557 PMC1088893

[B10] Gagliano M. Grimonprez M. Depczynski M. Renton M. (2017). Tuned in: plant roots use sound to locate water. *Oecologia* 184 151–160. 10.1007/s00442-017-3862-z 28382479

[B11] Gagliano M. Mancuso S. Robert D. (2012). Towards understanding plant bioacoustics. *Trends Plant Sci.* 17 323–325. 10.1016/j.tplants.2012.03.002 22445066

[B12] Ghosh R. Gururani M. A. Ponpandian L. N. Mishra R. C. Park S.-C. Jeong M.-J. (2017). Expression analysis of sound vibration-regulated genes by touch treatment in *Arabidopsis*. *Front. Plant Sci.* 8:100. 10.3389/fpls.2017.00100 28197168 PMC5281610

[B13] Ghosh R. Mishra R. C. Choi B. Kwon Y. S. Bae D. W. Park S.-C. (2016). Corrigendum: exposure to sound vibrations lead to transcriptomic, proteomic and hormonal changes in *Arabidopsis*. *Sci. Rep.* 6:37484. 10.1038/srep37484 27883000 PMC5122249

[B14] Grothe B. Pecka M. McAlpine D. (2010). Mechanisms of sound localization in mammals. *Physiol. Rev.* 90 983–1012. 10.1152/physrev.00026.2009 20664077

[B15] Hassanien R. H. Hou T. Z. Li Y. F. Li B. M. (2014). Advances in effects of sound waves on plants. *J. Integr. Agric.* 13 335–348. 10.1016/S2095-3119(13)60492-X

[B16] Hölttä T. Vesala T. Nikinmaa E. Perämäki M. Siivola E. Mencuccini M. (2005). Field measurements of ultrasonic acoustic emissions and stem diameter variations. New insight into the relationship between xylem tensions and embolism. *Tree Physiol.* 25 237–243. 10.1093/treephys/25.2.237 15574405

[B17] Hou T. Li B. Teng G. Zhou Q. Xiao Y. Qi L. (2009). Application of acoustic frequency technology to protected vegetable production. *Trans. Chin. Soc. Agric. Eng.* 25 156–160. 10.3969/j.issn.1002-6819.2009.2.030

[B18] Jeong M. J. Cho J. I. Park S. H. Kim K. H. Lee S. K. Kwon T.-R. (2014). Sound frequencies induce drought tolerance in rice plant. *Pak. J. Bot.* 46 2015–2020.

[B19] Jeong M. J. Shim C. K. Lee J. O. Kwon H. B. Kim Y. H. Lee S. K. (2008). Plant gene responses to frequency-specific sound signals. *Mol. Breed.* 21 217–226. 10.1007/s11032-007-9122-x

[B20] Kim J. Y. Ahn H. R. Kim S. T. Min C. W. Lee S. I. Kim J. A. (2016). Sound wave affects the expression of ethylene biosynthesis-related genes through control of transcription factors RIN and HB-1. *Plant Biotechnol. Rep.* 10 437–445. 10.1007/s11816-016-0419-2

[B21] Kim J.-Y. Lee J.-S. Kwon T.-R. Lee S.-I. Kim J.-A. Lee G.-M. (2015). Sound waves delay tomato fruit ripening by negatively regulating ethylene biosynthesis and signaling genes. *Postharvest Biol. Technol.* 110 43–50. 10.1016/j.postharvbio.2015.07.015

[B22] Kim T. H. Bohmer M. Hu H. Nishimura N. Schroeder J. I. (2010). Guard cell signal transduction network: advances in understanding abscisic acid, CO_2_, and Ca^2+^ signaling. *Annu. Rev. Plant Biol.* 61 561–591. 10.1146/annurev-arplant-042809-112226 20192751 PMC3056615

[B23] Kwon Y. S. Jeong M. J. Cha J. Jeong S. W. Park S. C. Shin S. C. (2012). Comparative proteomic analysis of plant responses to sound waves in Arabidopsis. *J. Plant Biotechnol.* 39 261–272. 10.5010/JPB.2012.39.4.261

[B24] Laschimke R. Burger M. Vallen H. (2006). Acoustic emission analysis and experiments with physical model systems reveal a peculiar nature of the xylem tension. *J. Plant Physiol.* 163 996–1007. 10.1016/j.jplph.2006.05.004 16872717

[B25] McCall R. P. (2010). “Sound speech and hearing,” in *Physics of the Human Body* ed. McCall R. P. (Baltimore, MD: JHU press) 116.

[B26] Meng Q. Zhou Q. Gao Y. Zheng S. Gao Y. (2012). Effects of plant acoustic frequency technology on the growth traits, chlorophyll content and endogenous hormones of *Lycopersicon esculentum*. *Hubei Agric. Sci.* 51 1591–1595.

[B27] Mishra R. C. Ghosh R. Bae H. (2016). Plant acoustics: in the search of a sound mechanism for sound signaling in plants. *J. Exp. Bot.* 67 4483–4494. 10.1093/jxb/erw235 27342223

[B28] Morales R. F. Seong K. M. Kim C. S. Jin Y. W. Min K. J. (2010). Effects of auditory stimuli on the lifespan of *Drosophila melanogaster*. *Entomol. Res.* 40 225–228. 10.1111/j.1748-5967.2010.00290.x

[B29] Qi L. Teng G. Hou T. Zhu B. Liu X. (2009). “Influence of sound wave stimulation on the growth of strawberry in sunlight greenhouse,” in *Computer and Computing Technologies in Agriculture* Vol. 317 eds Li D. L. Zhao C. J. (Stone Harbor, NJ: Springer) 449–454.

[B30] Qin Y. C. Lee W. C. Choi Y. C. Kim T. W. (2003). Biochemical and physiological changes in plants as a result of different sonic exposures. *Ultrasonics* 41 407–411. 10.1016/S0041-624X(03)00103-3 12788223

[B31] Riemann M. Dhakarey R. Hazman M. Miro B. Kohli A. Nick P. (2015). Exploring jasmonates in the hormonal network of drought and salinity responses. *Front. Plant Sci.* 6:1077. 10.3389/fpls.2015.01077 26648959 PMC4665137

[B32] Ritman K. T. Milburn J. A. (1990). Monitoring of ultrasonic and audible emissions from plants with or without vessels. *J. Exp. Bot.* 42 123–130. 10.1093/jxb/42.1.123

[B33] Shipman J. Wilson J. D. Higgins C. A. (2012). “Waves and Sound,” in *An Introduction to Physical Science* eds Shipman J. Wilson J. D. Higgins C. A. (Boston, MA: Cengage Learning) 134–142.

[B34] Theunissen F. E. Elie J. E. (2014). Neural processing of natural sounds. *Nat. Rev. Neurosci.* 15 355–366. 10.1038/nrn3731 24840800

[B35] Uematsu K. Suzuki N. Iwamae T. Inui M. Yukawa H. (2012). Increased fructose 1,6-bisphosphate aldolase in plastids enhances growth and photosynthesis of tobacco plants. *J. Exp. Bot.* 63 3001–3009. 10.1093/jxb/ers004 22323273

[B36] van Loon L. C. (1975). Polyacrylamide disc electrophoresis of the soluble leaf proteins from *Nicotiana tabacum* var. ‘Samsun’ and ‘Samsun NN’. IV. Similarity of qualitative changes of specific proteins after infection with different viruses and their relationship to acquired resistance. *Virology* 67 566–575. 10.1016/0042-6822(70)90395-8 18621356

[B37] Weinberger P. Measures M. (1979). Effects of the intensity of audible sound on the growth and development of Rideau winter wheat. *Can. J. Bot.* 57 1036–1039. 10.1139/b79-128

[B38] Yi J. Bochu W. Xiujuan W. Daohong W. Chuanren D. Toyama Y. (2003). Effect of sound wave on the metabolism of *Chrysanthemum* roots. *Colloids Surf. B Biointerfaces* 29 115–118. 10.1016/S0927-7765(02)00155-8 18243669

